# Comparison of PET/CT and PET/MR imaging and dosimetry of yttrium-90 (^90^Y) in patients with unresectable hepatic tumors who have received intra-arterial radioembolization therapy with ^90^Y microspheres

**DOI:** 10.1186/s40658-018-0222-y

**Published:** 2018-08-30

**Authors:** Karin Knešaurek, Abbas Tuli, Edward Kim, Sherif Heiba, Lale Kostakoglu

**Affiliations:** 1 Division of Nuclear Medicine, Department of Radiology, Icahn School of Medicine at Mount Sinai, One Gustave L. Levy Place, Box 1141, New York, NY 10029 USA; 2 Division of Interventional Radiology, Department of Radiology, Icahn School of Medicine at Mount Sinai, New York, USA

**Keywords:** ^90^Y PET/CT, ^90^Y PET/MRI, Dosimetry

## Abstract

**Background:**

The aim of our study was to compare ^90^Y dosimetry obtained from PET/MRI versus PET/CT post-therapy imaging among patients with primary or metastatic hepatic tumors.

First, a water-filled Jaszczak phantom containing fillable sphere with ^90^Y-chloride was acquired on both the PET/CT and PET/MRI systems, in order to check the cross-calibration of the modalities. Following selective internal radiation therapy (SIRT) with ^90^Y microspheres, 32 patients were imaged on a PET/CT system, immediately followed by a PET/MRI study. Reconstructed images were transferred to a common platform and used to calculate ^90^Y dosimetry. A Passing-Bablok regression scatter diagram and the Bland and Altman method were used to analyze the difference between the dosimetry values.

**Results:**

The phantom study showed that both modalities were calibrated with less than 1% error. The mean liver doses for the 32 subjects calculated from PET/CT and PET/MRI were 51.6 ± 24.7 Gy and 46.5 ± 22.7 Gy, respectively, with a mean difference of 5.1 ± 5.0 Gy. The repeatability coefficient was 9.0 (18.5% of the mean). The Spearman rank correlation coefficient was very high, *ρ* = 0.97. Although the maximum dose to the liver can be significantly different (up to 40%), mean liver doses from each modalities were relatively close, with a difference of 18.5% or less.

**Conclusions:**

The two main contributors to the difference in ^90^Y dosimetry calculations using PET/CT versus PET/MRI can be attributed to the differences in regions of interest (ROIs) and differences attributed to attenuation correction. Due to the superior soft-tissue contrast of MRI, liver contours are usually better seen than in CT images. However, PET/CT provides better quantification of PET images, due to better attenuation correction. In spite of these differences, our results demonstrate that the dosimetry values obtained from PET/MRI and PET/CT in post-therapy ^90^Y studies were similar.

## Background

Yttrium-90 (^90^Y) microsphere selective internal radiation therapy (SIRT) is emerging as a promising treatment modality in the management of patients with unresectable primary or metastatic hepatic tumors [1, 2]. The true ^90^Y distribution and dosimetry can only be obtained post-therapy using bremsstrahlung SPECT (bSPECT), PET/CT, or PET/MRI imaging [3]. The importance of post-therapy ^90^Y imaging is twofold. First, it is used for detection of possible extrahepatic activity, which can cause serious complications, such as ulceration and GI bleeds [4–6]. Second, post-therapy quantitative ^90^Y imaging can be used to estimate the absorbed radiation dose delivered to liver tumors and normal liver tissue. These data can help us to determine whether patients’ adverse events, treatment successes, or treatment failures can be attributed to the dose that the tumor or normal liver received; they are also expected to be an important predictor of treatment efficacy [7].

Quantitative bremsstrahlung imaging is challenging due to scatter, septal penetration, the continuous nature of the bremsstrahlung energy spectrum, and inefficient bremsstrahlung production [8]. Post-therapy PET/CT or PET/MRI ^90^Y images are far superior, both qualitatively and quantitatively than bSPECT ^90^Y images [9]. In this prospective analysis, ^90^Y dosimetry calculations from PET/CT were compared with those from PET/MRI. PET/CT was used as the gold standard because it is the established method with accurate attenuation correction; PET/MRI attenuation correction method has yet to be optimized.

## Methods

**Fig. 1:**
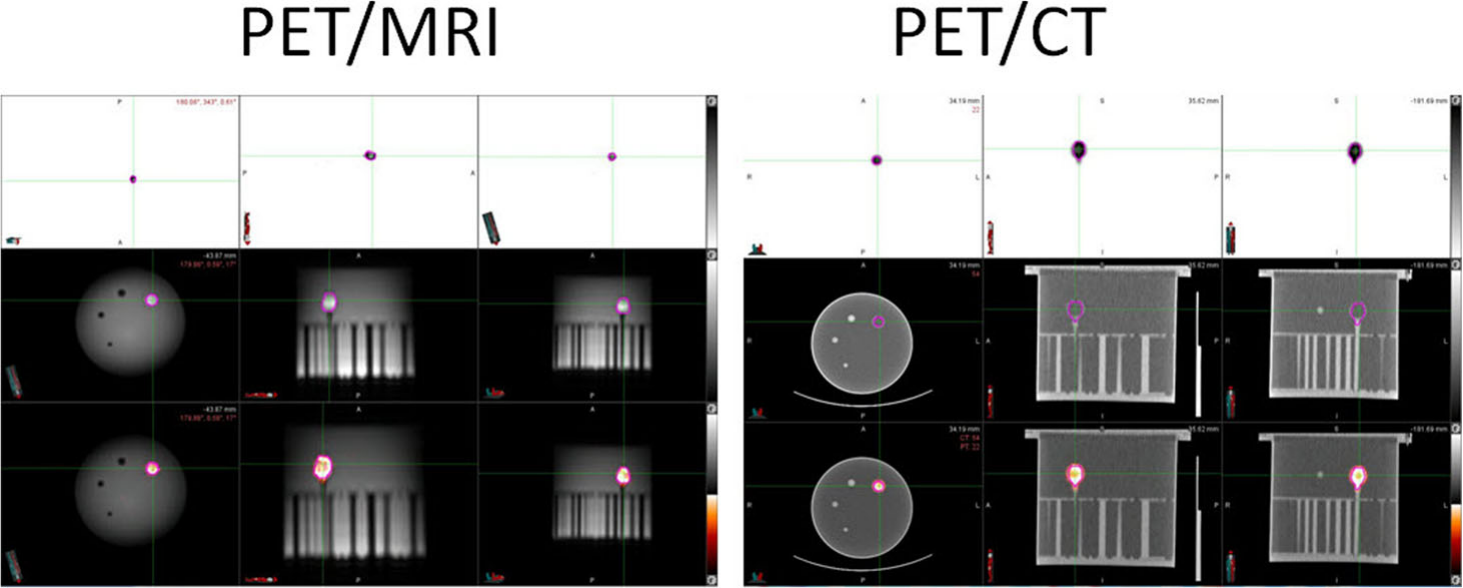
PET/MRI and PET/CT images of the Jaszczak water-filled phantom with a 24.8-mm fillable sphere, filled with 1147.0 MBq of ^90^Y-chloride. In the first row, there are PET images, in the second row MRI and CT images, respectively, and in the third row fused PET/MRI and PET/CT images, respectively. The results of the phantom study showed that these two systems are very closely calibrated for ^90^Y, i.e., less than 1% difference

**Fig. 2:**
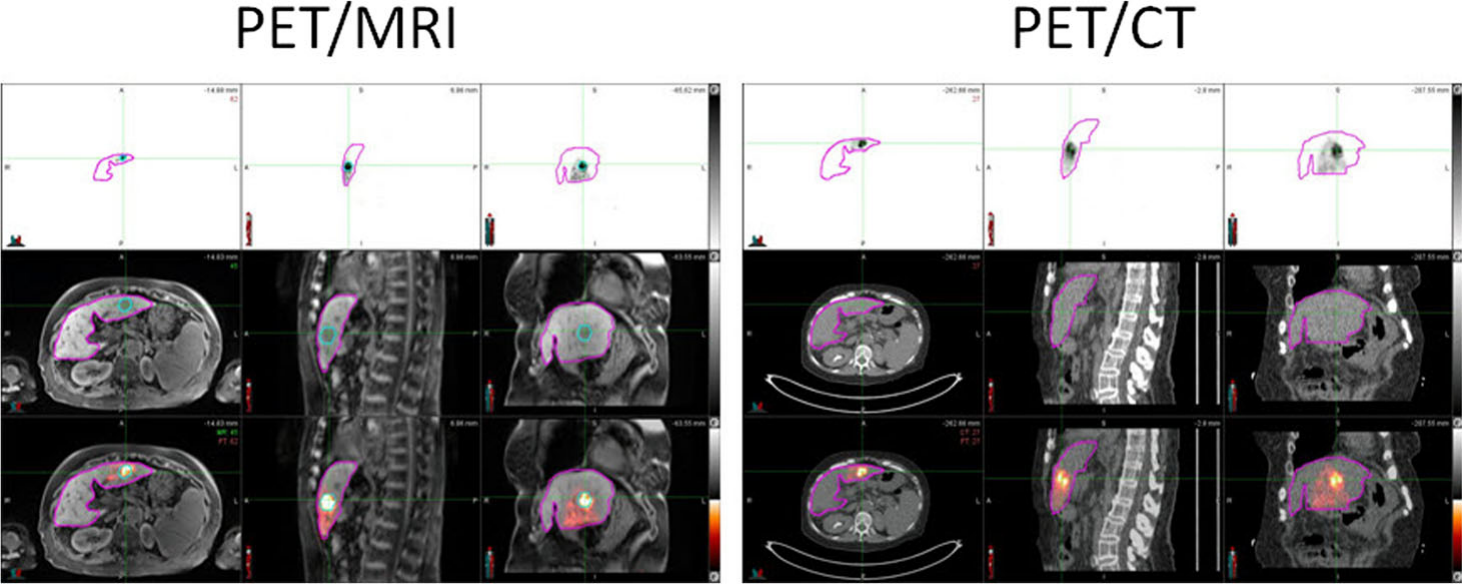
PET/MRI and PET/CT images of the subject with the highest mean liver dose difference of 18.5%. In the first row, there are PET images, in the second row MRI and CT images, respectively, and in the third row fused PET/MRI and PET/CT images, respectively. Due to different field of view and voxel sizes in PET/MRI and PET/CT, the size of the images is different for different modalities and different studies. However, the distribution of ^90^Y microspheres is practically identical. The mean liver dose obtained from PET/CT was 38.81 Gy and 31.64 Gy from PET/MRI. In PET/MRI image, left image, second row, one can clearly see the tumor and tumor ROI almost perfectly matches the most intense ^90^Y microsphere distribution

In a prospective study, after SIRT with ^90^Y microspheres, 32 patients were imaged on a four-ring, time-of-flight (TOF), PET/CT system Biograph mCT (Siemens Medical Systems, Erlangen, Germany). The PET properties of the system are given in Table 1. The low mA, non-diagnostic CT images were used for attenuation correction and localization of the ^90^Y microspheres in the PET/CT studies. The acquisition time was 15 min. The reconstruction matrix size was 200 × 200 × 75 and voxel size 4.07 × 4.07 × 3.00 mm^3^. Immediately after the PET/CT study, a PET/MRI study was done on a 3 T Biograph mMR scanner (Siemens Medical Systems, Erlangen, Germany). The mMR system uses avalanche photo diodes (APDs) instead of photomultipier tubes and does not have TOF capabilities. PET properties of the mMR system are also given in Table 1. The acquisition time was 40 min, and attenuation correction was done using four-tissue segmentation (air, lung, fat, soft tissue) of two-point Dixon sequence [10]. The reconstruction matrix size was 172 × 172 × 127 and voxel size 4.17 × 4.17 × 2.03 mm^3^. For liver delineation and creation of ROIs, usually AX VIBE PRE sequence was used although, we also acquired COR T2 HASTE, AX HASTE, AX T2 FS, and some other sequences related to motion correction using 2D MRI navigator [11]. For both modalities, due to relatively long axial field of views (Table 1), only one-bed position acquisitions were used. Both, mCT and mMR use the same model-based scatter correction [12, 13], although the scatter scaling is slightly different in the two scanners. In ^90^Y imaging, the number of true events is low and the LSO and bremsstrahlung random coincidences are often a large fraction of the prompt coincidences [14]. Therefore, it is a necessity to apply a smoothing technique on the measured delayed coincidences. This is possible only if separate prompt and random events are acquired. Consequently, both mCT and mMR use the delay window to measure randoms. By proper smoothing of randoms, it is possible to reduce or eliminate sinogram bins in which randoms (because of noise) are higher than prompts. However, randoms should not be directly “subtracted” in the iterative update equation, but one should use an algorithm where randoms are added to estimate of the trues in the update equation. Such an algorithm is 3D ordinary Poisson-ordered subset expectation maximization (OP-OSEM3D) in conventional and TOF mode (OP-OSEM3D+TOF), and OP-OSEM3D with point spread function correction (OP-OSEM3D+PSF) in conventional and TOF mode (OP-OSEM3D+PSF+TOF) [15]. TOF reconstruction deals better with randoms because the TOF kernel tends to filter out all events with the spatial position of the source outside the patient. Typically, randoms have a TOF that is not correlated with the actual position of a source in a line of response (LOR). This also increases the signal-to-noise ratio (SNR) [16, 17]. However, the random correction implemented in mCT with TOF and in mMR without TOF appears to work well in ^90^Y studies, even at high bremsstrahlung and LSO random coincidences [15, 18]. mCT gain in SNR due to TOF and consequently, in measured sensitivity, is compensated in mMR by longer axial FOV (Table 1) and smaller bore diameter. Contrast was not used for neither CT nor MRI images, because of three reasons. First, we could not get hospital approval for using contrast media with possible adverse reactions in our research. Second, costs of contrast media, especially for MRI, are very high and we did not have funds to cover these costs. The third reason was that all of these subjects participating in the study were terminally ill patients and already exhausted from ^90^Y-SIRT procedure, when they were approached to volunteer for this study. The research imaging protocol had to be totally non-invasive, i.e., without any injections or administrations of contrast in order to have sufficient number of volunteers. The data were reconstructed according to results of multi-institutional phantom ^90^Y PET/CT and PET/MRI studies. The purpose of these studies was to find optimal acquisition and reconstruction parameters for ^90^Y post-therapy PET/CT and PET/MRI imaging. The studies were called QUEST [18] and MR-QUEST [11, 19] study, respectively. Consequently, PET images obtained on mCT PET/CT system were reconstructed with a CT-based attenuation correction, using the OP-OSEM3D+TOF+PSF with two iterations, 21 subsets (2i21s), and 5-mm Gaussian post-reconstruction filter. For PET images obtained on PET/MRI system, we used OP-OSEM3D+PSF reconstructed algorithm with three iterations and 21 subsets (3i21s) and 5-mm Gaussian post-reconstruction filter. In addition to clinical studies, a Jaszczak water-filled phantom with a 24.8-mm fillable sphere filled with 1147.0 MBq of ^90^Y-chloride was acquired on both modalities, i.e., PET/CT and PET/MRI, using the same acquisition and processing parameters (Fig. 1) as used in clinical studies. The purpose of the phantom study was to check cross-calibration between mCT and mMR systems. Both systems are calibrated daily with ^68^Ge cylinder sources provided by the vendor, with NIST traceable activities. Reconstructed images were transferred to a common platform and used to calculate ^90^Y dosimetry using MIM 6.6 software (MIM Software Inc.) (Fig. 2). Local deposition method (LDM) with known activity of ^90^Y was used to calculate dosimetry [20]. Because ^90^Y decays primarily with β^−^-emission (mean 0.937 MeV, 64.2 h half-life, 2.5 mm mean tissue penetration, 11 mm max tissue penetration) [11], LDM seems to be a practical alternative to a more complicated dose-point kernel (DPK) convolution approach. Some results even suggested that in certain cases, the LDM will out-perform conventional DPK convolution in post-radioembolization ^90^Y dosimetry based on PET/CT or PET/MRI imaging [20].

**Table 1:** PET system characteristics for mCT (PET/CT) and mMRI (PET/MRI) system

	mCT	mMR
Crystal material	LSO	LSO
Crystal element dimension	4 × 4 × 20 mm	4 × 4 × 20 mm
Detector ring diameter	780 mm	656 mm
Transaxial FoV	680 mm	588 mm
Axial FoV	216 mm	258 mm
Coincidence window	4.1 ns	4.1 ns
System energy resolution	≤ 12% FWHM	≤ 14% FWHM
System time resolution	540 ps typical	N/A (no TOF system)
Sensitivity (cps/kBq)	10.2	14.1
Spatial resolution—transverse	FWHM at 1 cm (mm) 4.5	FWHM at 1 cm (mm) 4.2
Spatial resolution—axial	FWHM at 1 cm (mm) 4.7	FWHM at 1 cm (mm) 4.6
Peak NEC rate (kcps)	180 at ≤ 28 kBq/cc	180 at ≤ 26 kBq/cc
Scatter fraction (at peak NEC rate)	37%	38%
Reconstruction parameters	PSF-TOF-OSEM 2i21s	PSF-OSEM 3i21s

### Statistical analyses

**Fig. 3:**
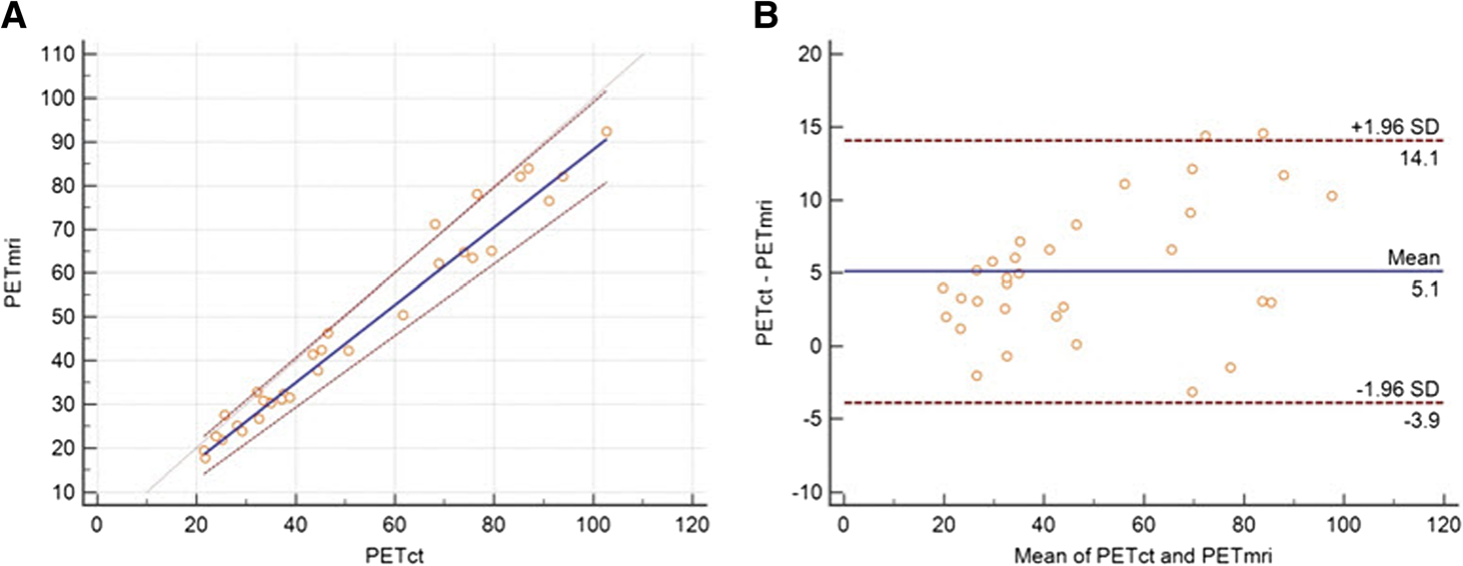
**a** The Passing-Bablok regression scatter diagram with the regression line (solid line), the confidence interval for regression line (dashed lines), and identity line (*x* = *y*, dotted line), for mean liver dose values in Gy obtained from both methods and all 32 subjects. PETct denotes mean liver dose in Gy obtained using PET/CT imaging, and PETmri denotes mean liver dose in Gy obtained using PET/MRI imaging (*n* = 32, *ρ* = 0.97). **b** Blant-Altman plot for all 32 subjects, with a mean difference of 5.1 ± 5.0 Gy. The repeatability coefficient was 0.15 (12.3% of the mean)

The Passing-Bablok regression scatter diagram with the regression line (solid line), the confidence intervals for the regression line (dashed lines), and identity line (*x* = *y*, dotted line), (Fig. 3) were used to compare dosimetry values obtained by both methods [21]. A Spearman rank correlation coefficient (*ρ*) was also reported. The Bland and Altman method [22] was used to analyze the difference between dosimetry values obtained with these two approaches and to test repeatability of these results. The repeatability coefficient was calculated as 1.96 times the SD of the differences [23]. The dosimetry data was reported as mean ± SD. For comparison, the repeatability coefficient was also given as a percentage of the average values of the doses obtained by these two approaches. The statistical analysis was performed using MedCalc Software bvba, 17.8.6—64 bit version. The differences between PET/CT and PET/MRI dosimetry values were expressed as percentage of PET/CT values, because as mentioned above, PET/CT was used as a gold standard method, with established attenuation correction and quantification.

## Results

The phantom study (Fig. 1) showed that both systems provided very close volume and activity concentration values. For fillable sphere volume, mCT gave 9.97 ml and mMR gave 10.08 ml, respectively. The true value is 8.0 ml, but due to scatter, both modalities overestimated volume. For activity concentration, the values were 115.0 MBq/ml and 113.8 MBq/ml for mCT and mMR, respectively. These results show that in our studies, these two systems are very closely calibrated for ^90^Y, i.e., less than 1% difference. However, the phantom study does not suffer from motion artifacts due to breathing, or from errors in attenuation correction, because the phantom has a uniform shape and it does not contain any bony structures.

The clinical comparison was performed on 32 subjects, 25males and 7 females, with mean age of 64.7 ± 9.9 (mean ± SD) years. The imaging protocols were approved by the institutional review board, and written informed consent was obtained for each subject enrolled. Twenty-five patients were treated with TheraSphere ® (glass microspheres; BTG, London, UK) and the rest of 7 subjects with SIR-Sphere® (resin microspheres; Sirtex Medical, Sydney, Australia). Majority of patients, 24 of them, had hepatocellular carcinoma (HCC) and 4 had primary colorectal cancer (CRC) and there were one case each of liver metastases from primary breast (MAM), neoendocrine tumor (NET), and thyroid and pancreatic cancer.

For PET/CT and PET/MRI modalities, the mean liver dose for all 32 subjects was 51.6 ± 24.7 Gy and 46.5 ± 22.7 Gy, respectively, with a mean difference of 5.1 ± 5.0 Gy. The repeatability coefficient was 9.0 (18.5% of the mean). The Spearman rank correlation coefficient was very high, *ρ* = 0.97 (Fig. 3). Although the maximum dose to the liver can be significantly different, up to 40%, mean liver dose from both modalities was relatively close, with a maximum difference of 18.5%. In four patients, PET/MRI gave close, but slightly higher dose. In these four subjects, the dose difference was less than 8%. In all other subjects, PET/CT gave higher doses than PET/MRI. The differences were in the range of 18.5 to 2.4%. PET/CT values ranged from 102.7 to 21.4 Gy, and PET/MRI values ranged from 92.4 to 17.7 Gy. These maximum and minimal PET/CT and PET/MRI dose values were obtained on the same subjects.

## Discussion

To the best of our knowledge, this is the first comparison of PET/CT and PET/MRI imaging and dosimetry in ^90^Y post-treatment studies [3]. In mutual comparison between PET/CT and PET/MRI, each modality has advantages and disadvantages. The advantages of PET/CT modality is better attenuation correction and thus better quantification, plus faster acquisition time. In PET/CT, the time for CT acquisition is measured in seconds and total acquisition time is determined by the PET component. In PET/MRI, it is the other way around. MRI determines acquisition time, which is mostly determined by the number of MRI sequences. However, the advantages of PET/MRI are lower radiation dose and better soft-tissue contrast, which is essential for accurately delineating healthy liver tissue versus tumors during analysis [24]. Also, due to their different data acquisition approaches, i.e., sequential for PET/CT vs. simultaneous for PET/MRI, PET/MRI offers the opportunity to directly image respiration liver motion during the PET acquisition and correct for it during the PET reconstruction [25]. In addition, high spatial resolution MR images, which offer high soft-tissue contrast, could be used in partial volume (PV) correction of the lower resolution PET images [26, 27].

**Fig. 4:**
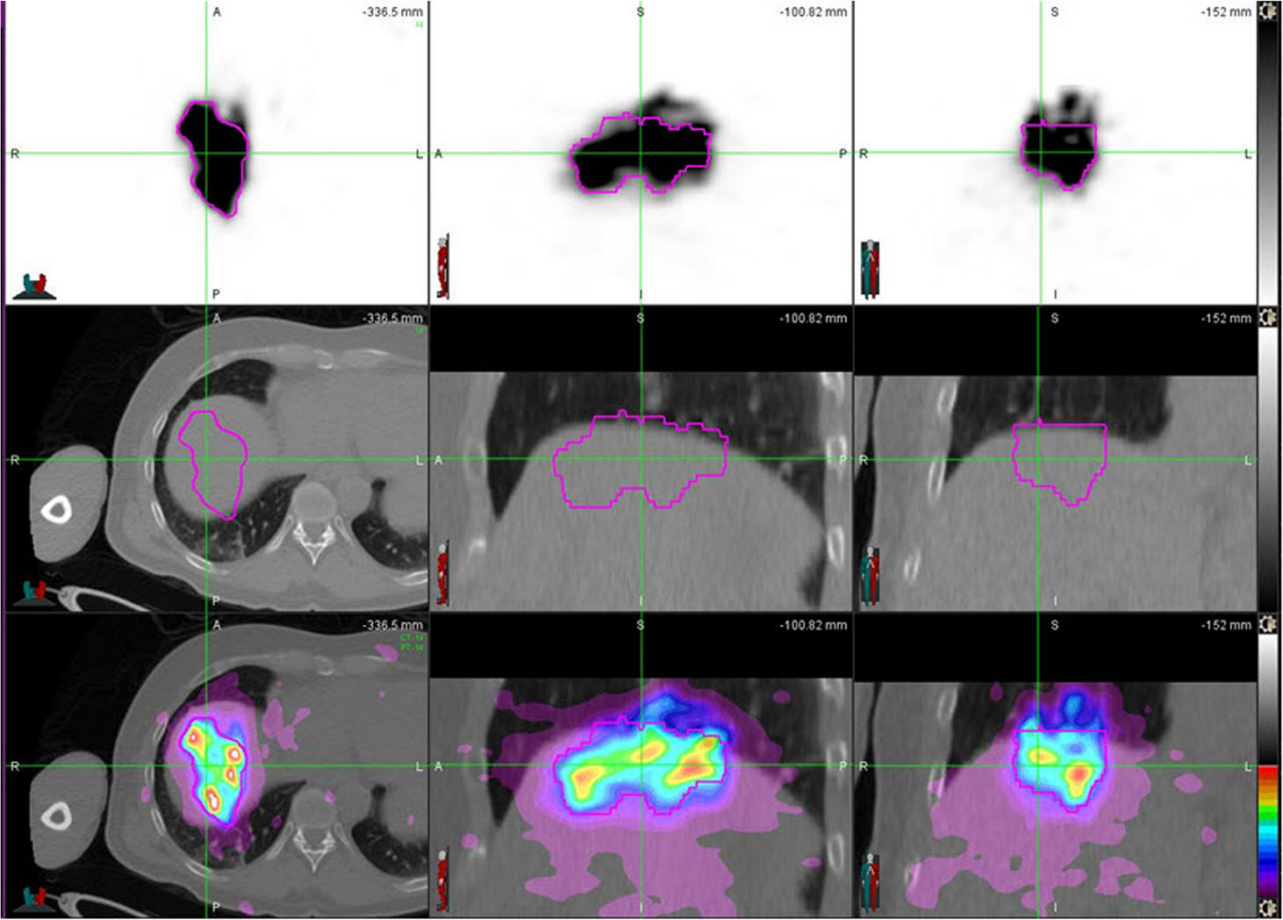
First row shows PET ^90^Y images, second row corresponding CT images, and third row fused PET/CT images. In fused coronal and sagittal PET/CT images, one can clearly see ^90^Y spillover into lungs due to respiratory motion artifacts

In our study, PET/MRI led to underestimation of the mean liver dose values by less than 10% on average, when compared to PET/CT. In some cases, PET/MRI values were almost the same or even slightly higher than PET/CT values. In our previous work [28], in which we compared MR-based and CT-based attenuation corrections on the same subjects, SUVmean and SUVmax values obtained from PET/CT were slightly higher in values than the corresponding values obtained from PET/MRI. It seems that the same trend is present in comparison of dosimetry values in ^90^Y post-therapy studies (Fig. 3). In both cases, the reason for the variation in these values lies in the difference of attenuation corrections applied. However, in this comparison, additional source of difference is also attributed to creation of ROIs from CT and MRI anatomic images, which were used for dosimetry calculations. In Fig. 2, one can see very small difference between PET/MRI and PET/CT ROIs. However, numerical results shows that liver volume determinate from PET/CT was 858.35 cm^3^ and from PET/MRI 801.28 cm^3^. This difference in volume determination and consequently the mass of liver, which is calculated by multiplying volume in cubic centimeter to 0.00103 kg/cm^3^, has great impact on dosimetry calculations. LDM assumes that all of the energy released by the ^90^Y beta-particle decay remains within the same voxel. Using the average energy of beta particles, the total energy deposited per unit volume over the entire isotope decay, which is assumed to be infinity due to the permanent implant of microspheres, can be calculated. Calculations give that in each voxel we can assume that the dose in Gy is equal to product of activity in GBq × 49.38/mass (kg) [29]. Here, we used corrected activity for any extra-hepatic distribution such as lung shunting and corrected for residual activity. In this particular case, where tumor was clearly visible in MRI images, we calculated tumor-to-normal tissue (T/N) dose ratio. For this purpose, we used MRI image with tumor ROI to merge with CT images from PET/CT, using deformable transformation provided by MIM software. Although it is beyond the scope of this paper, the T/N ratio in this particular case was 24.90 for PET/CT and 30.00 for PET/MRI study. However, both modalities resulted in a tumor volume about 7.0 cm^3^ and for such small tumors partial volume effects would greatly affect quantification and dosimetry calculations. The limitation of our approach was that we did not use contrast media in CT nor MRI images. Without contrast media, delineation of lesions and tumors in liver is difficult and not always accurate. Intra hepatic dosimetry, like calculations of T/N ratios, requires using of contrast in anatomical modalities, as well as, PV corrections for lesions smaller than 2.5 cm [19]. Also, for lesions in superior hepatic lobes (Fig. 4), respiratory motion effect can alter ^90^Y imaging and dosimetry and motion correction should be applied [25].

In clinical settings, in most places around the world, dosimetry related to SIRT with ^90^Y microspheres is done using simple software provided by vendors. When SIR-Spheres are used, the body surface area (BSA) method is mostly used according which, activity A(GBq) = (BSA − 0.2) × (tumor volume/total liver volume). For TheraSpheres, the LDM is used for total liver or lobe in treatment., i.e., activity A(GBq) is given as a product of dose in Gy multiplied by mass (kg) and divided by 49.38 [29], where a typical dose between 100 and 120 Gy is selected for TheraSphere treatments involving patients with HCC. The target dose for a particular solid tumor is not known, but it is currently believed that this dose range balances the response rate with the risk of hepatic fibrosis.

However, in our study, we have used more sophisticated voxel-based dosimetry calculations, which are providing iso-dose curves and dose-volume histograms (DVH). Such advanced and personalized dosimetry approaches are not reimbursable in many countries, including USA, and these studies are still in research domain only. The optimal software should have good segmentation routine for easy delineation of the liver, other organs, and structures and provide accurate dosimetry calculations for all these volume of interests (VOIs), including iso-dose curves, DVH, minimal, maximal, and average doses. The results also should be easily exported to reports and spreadsheets for further evaluations and comparisons. The same software can also be used in pre-treatment dose estimations, using ^99m^Tc macroaggregated albumin (MAA), mimicking ^90^Y distribution. However, using MAA to predict ^90^Y distribution is still an approximation. We believe that we were the first to report that ^90^Y distribution does not always follow the MAA distribution and that in some situations, there can be large discrepancies between these distributions [30]. Other group went even further and concluded that MAA is not good predictor of ^90^Y distribution at all [31]. Our opinion is that MAA is useful in predicting ^90^Y distributions, but the final ^90^Y distribution can only be confirmed by post-therapy imaging using bSPECT, or even better, using PET/CT or PET/MRI. The main source of MAA and ^90^Y distribution mismatch, in our experience [32], is attributed to catheter positioning. The role of interventional radiologists is essential in that regard, i.e., in positioning the catheter and avoiding stealing artery branches and critical bifurcations.

Treatment of patients with unresectable primary or metastatic hepatic tumors with ^90^Y microsphere SIRT continues to develop at a rapid pace. Overcoming technical angiographic challenges, clinical research is expanding indications in many different tumor types. However, fine tuning of ^90^Y dosimetry and optimizing quantitative imaging in daily practice is still essential. We strongly believe that PET/CT and PET/MRI can fulfill that role of image-based accurate ^90^Y imaging and dosimetry.

## Conclusions

Two main sources of ^90^Y dosimetry difference between PET/CT and PET/MRI calculations can be attributed to the differences in ROIs and differences attributed to attenuation correction. Due to better MRI soft-tissue contrast, liver contours are usually better seen in MRI images. However, PET/CT provides better quantification of PET images, due to better attenuation correction. In spite of these differences, our results demonstrate that the dosimetry values obtained from PET/MRI and PET/CT in post-therapy ^90^Y studies were similar.

## Abbreviation

^90^Y: Yttrium-90
bSPECT: Bremsstrahlung SPECT
DPK: Dose-point kernel
LDM: Local deposition method
SIRT: Selective internal radiation therapy
